# 

**Published:** 1831-08

**Authors:** 


### MONTHLY LIST OF MEDICAL BOOKS.

[*Medical Works cannot be entered on this List unless a copy be sent for the purpose, the titles of Books having frequently been sent to us as published which have not been published for weeks, or even months, after.*]

Essays and Orations read and delivered at the Royal College of Physicians: to which is added, an Account of the Opening of the Tomb of King Charles I. By Sir Henry Halford, Bart. M.D. G.C.H., President of the College.—8vo. pp. 192. Murray, London.

The Art of Cupping; being a brief History of the Operation, from its Origin to the present Time; its Utility; minute Rules for its Performance; a List of the Diseases in which it is most beneficial; and a Description of the various Instruments employed, &c. By G. F. Knox, Cupper at the Westminster Hospital, &c.—8vo. pp. 68. Highley, London.

The Veterinarian: from the commencement to the last Number. Published Monthly. Edited by Messrs. Percivall and Youatt. Longman and Co.

A Practical Treatise on Diseases of the Head.—8vo. pp. 121. Dublin.

Dublin Medical Transactions; a Series of Papers by Members of the Association of Fellows and Licentiates of the King and Queen's College of Physicians in Ireland. New Series. Vol. I. Part I.—8vo. pp. 384; plates. Leckie, Dublin.

Outlines of Physiology: with an Appendix, containing Heads of Lectures on Pathology and Therapeutics. By William Pulteney Alison, M.D. F.R.S.E., Fellow of the Royal College of Physicians, and Professor of the Institutes of Medicine in the University of Edinburgh.—8vo. pp. 452. Blackwood, Edinburgh; Cadell, London.

An Attempt to simplify the Treatment of Sexual Diseases. By James Thorn, Member of the Royal College of Surgeons, London.—8vo. pp. 240. Highley, London.

First Principles of Medicine. By Archibald Billing, M.D., Fellow of the Royal College of Physicians, Lecturer on the Theory and Practice of Medicine, &c.—8vo. pp. 131. Underwood, London.

History of the Epidemic Spasmodic Cholera of Russia; including a copious Account of the Disease which has prevailed in India, and which has travelled, under that name, from Asia into Europe. Illustrated by numerous Official and other Documents, explanatory of the Nature, Treatment, and Prevention of the Malady. By Bisset Hawkins, M.D., Fellow of the Royal College of Physicians, &c.—8vo. pp. 306. Murray, London.

